# Effect of Process Temperature on Density and Electrical Characteristics of Hf_0.5_Zr_0.5_O_2_ Thin Films Prepared by Plasma-Enhanced Atomic Layer Deposition

**DOI:** 10.3390/nano12030548

**Published:** 2022-02-05

**Authors:** Hak-Gyeong Kim, Da-Hee Hong, Jae-Hoon Yoo, Hee-Chul Lee

**Affiliations:** Department of Advanced Materials Engineering, Korea Polytechnic University, Siheung 15073, Korea; hakkyung5280@hanmail.net (H.-G.K.); ghdekgml9768@naver.com (D.-H.H.); dbwogns96@gmail.com (J.-H.Y.)

**Keywords:** HZO, PEALD, ferroelectric memory, deposition temperature, film density, remanent polarization, fatigue endurance

## Abstract

Hf*_x_*Zr_1−*x*_O_2_ (HZO) thin films have excellent potential for application in various devices, including ferroelectric transistors and semiconductor memories. However, such applications are hindered by the low remanent polarization (P_r_) and fatigue endurance of these films. To overcome these limitations, in this study, HZO thin films were fabricated via plasma-enhanced atomic layer deposition (PEALD), and the effects of the deposition and post-annealing temperatures on the density, crystallinity, and electrical properties of the thin films were analyzed. The thin films obtained via PEALD were characterized using cross-sectional transmission electron microscopy images and energy-dispersive spectroscopy analysis. An HZO thin film deposited at 180 °C exhibited the highest o-phase proportion as well as the highest density. By contrast, mixed secondary phases were observed in a thin film deposited at 280 °C. Furthermore, a post-annealing temperature of 600 °C yielded the highest thin film density, and the highest 2P_r_ value and fatigue endurance were obtained for the film deposited at 180 °C and post-annealed at 600 °C. In addition, we developed three different methods to further enhance the density of the films. Consequently, an enhanced maximum density and exceptional fatigue endurance of 2.5 × 10^7^ cycles were obtained.

## 1. Introduction

Since the report of ferroelectric behavior in HfO_2_-based thin films, studies have been conducted on HfO_2_ thin films doped with different elements. Particularly, Hf*_x_*Zr_1−*x*_O_2_ (HZO) thin films, which exhibit ferroelectricity even at thicknesses of a few nanometers, have gained increasing attention [1]. Among the diverse types of available ferroelectric materials, metal oxides have attained considerable technological importance owing to their compatibility with current complementary metal–oxide–semiconductor (CMOS) technology as well as large-scale integration. Therefore, active research has been underway for the application of HZO thin films to a variety of devices such as ferroelectric transistors, synapse devices, and ferroelectric tunneling junctions [2,3,4,5,6].

For the practical application of HZO thin films to semiconductor memories, it is necessary to overcome the issues of remanent polarization (P_r_) and fatigue endurance. Pb(Zr,Ti)O_3_-based materials having the crystal structure of perovskites, which are ferroelectric materials that have been studied extensively, exhibit a low wake-up effect, and show stable characteristics with exceptional fatigue endurance over 10^10^ cycles [7,8,9]. In this context, studies on improving the properties of HZO have been actively underway. In particular, research on the effects of crystal structure, oxygen defects inside thin films, grain size, and interface engineering using electrodes on changes in electrical properties has been mainly reported [10,11,12,13,14]. HZO thin films have a variety of crystalline phases such as tetragonal (t-, P4_2_/nmc), monoclinic (m-, P2_1_/c), and orthorhombic (o-, Pca2_1_) phases, of which the o-phase exhibits ferroelectric properties. However, the m-phase has been generally reported to be the stable phase of HZO [10,15], and research has been conducted to achieve a high ratio and stability of the o-phase in HZO films [16,17,18].

For HZO deposition, thermal atomic layer deposition (THALD) is mainly used. Moreover, there has been insufficient investigation on the properties of HZO thin films deposited by plasma-enhanced atomic layer deposition (PEALD) [19]. PEALD is capable of the high-density deposition of thin films and has the advantage of enabling low-temperature deposition [20,21,22]. The regions wherein the o-, t-, and m-phases of HZO are formed vary depending on grain size and temperature. Therefore, density improvement in the deposition process is expected through the stabilization of the o-phase and increasing the grain size through low-temperature deposition using PEALD [10,23]. In addition, to the best of the authors’ knowledge, there is no report on the relationship between the PEALD process temperature and changes in the electrical properties with respect to HZO density.

In this study, the initial process conditions for fabricating PEALD HZO thin films were set according to the deposition temperature, and the effect of the deposition temperature on the density and crystallinity of the thin films was analyzed. In addition, the optimal conditions for fabricating HZO thin films by PEALD were derived by examining the thin film density and crystallinity according to the post-annealing temperature. Furthermore, the effects of the variation of the HZO thin film density with the process temperature on the crystallinity of the o-phase exhibiting ferroelectricity, as well as on the electrical characteristics such as polarization hysteresis loops (P-E loops) and fatigue endurance, were investigated. Finally, process improvement methods for obtaining HZO thin films with high density and excellent electrical properties at a low deposition temperature of 100 °C were determined, and the results were comparatively analyzed.

## 2. Materials and Methods

### 2.1. HZO Thin Film Deposition by PEALD

**Figure 1:**
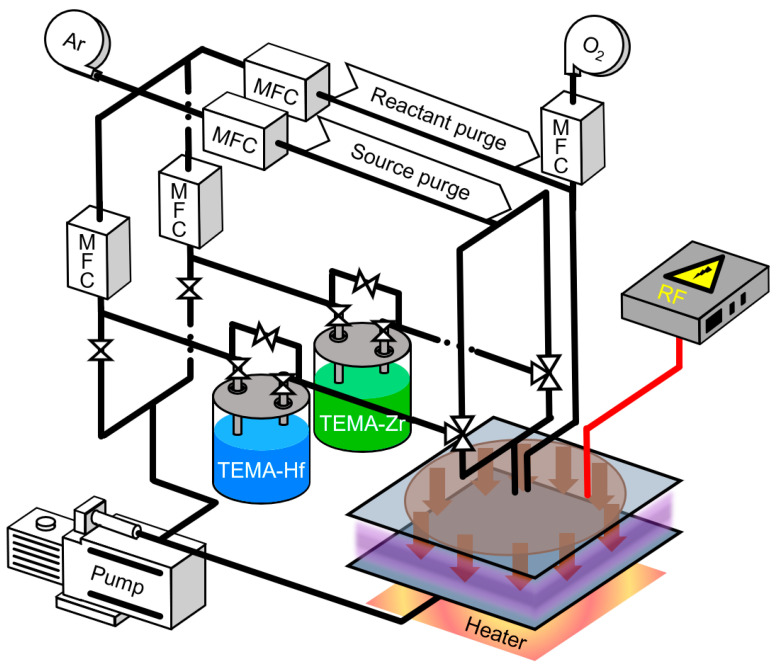
Schematic of PEALD setup used in this study.

For HZO thin film deposition, a substrate comprising a 50 nm TiN bottom electrode deposited on a SiO_2_(100 nm)/Si wafer was used. HZO thin film deposition was performed by PEALD (iOV-dx2, iSAC research, Hwaseong, Korea) using the experimental setup illustrated in Figure 1, and tetrakis(ethylmethylamido)-hafnium (TEMA-Hf, iChems, Hwaseong, Korea) and tetrakis(ethylmethylamido)-zirconium (TEMA-Zr, iChems, Hwaseong, Korea) were used as the precursors of HfO_2_ and ZrO_2_, respectively. To fabricate the HZO thin films, HfO_2_ and ZrO_2_ were alternately deposited in a 1:1 ratio, and this cycle was repeated until a thin film with a thickness of 10 nm was obtained. To obtain an optimal HZO thin film, deposition was performed in a temperature range of 100 to 280 °C. O_2_ gas was injected as a reactant, and oxidation was induced through a 200 W plasma discharge to form oxides. The detailed PEALD process conditions are outlined in Table 1. To prepare the top electrode for the evaluation of the electrical properties, a shadow mask was used, and a TiN electrode with a diameter of 200 μm was deposited at a thickness of 50 nm by reactive sputtering. Next, as shown in Table 2, crystallization of the HZO thin films was performed by post-annealing using rapid thermal annealing (RTA). Post-annealing was performed for 30 s in a temperature range of 500 to 700 °C in a nitrogen ambient of 5 Torr.

### 2.2. Characterization of HZO Thin Films

The thickness and refractive index of the deposited single oxides of HfO_2_ and ZrO_2_ and the HZO thin films were evaluated using an ellipsometer (Elli-SE, Ellipso technology, Suwon, Korea). The shape of the thin film cross-section and the elemental composition were analyzed using transmission electron microscopy (TEM) (NEO ARM, JEOL, Tokyo, Japan) and energy-dispersive spectroscopy (EDS) (JED-2300T, JEOL, Tokyo, Japan), respectively. The crystalline structure of the HZO thin films was measured by high-resolution X-ray diffraction (HR-XRD) (Smartlab, Rigaku, Tokyo, Japan) in Bragg–Brentano geometry, and the density of the thin film was calculated through X-ray reflectometry (XRR) analysis on the same instrument. Electrical properties such as the P-E curve and fatigue endurance of the thin film were evaluated using a TF analyzer (TF-2000E, aixACCT, Aachen, Germany) connected to a microprobe station (APX-6B, WIT, Suwon, Korea). Hysteresis loop measurements were performed at a frequency of 1 kHz with a triangle pulse of ±3 V. Fatigue endurance measurements were conducted by the continuous application of a square pulse of ±3 V at 10 kHz along with a 1 kHz triangle pulse that was applied five times at each time point to measure remnant polarization.

## 3. Results and Discussion

**Figure 2:**
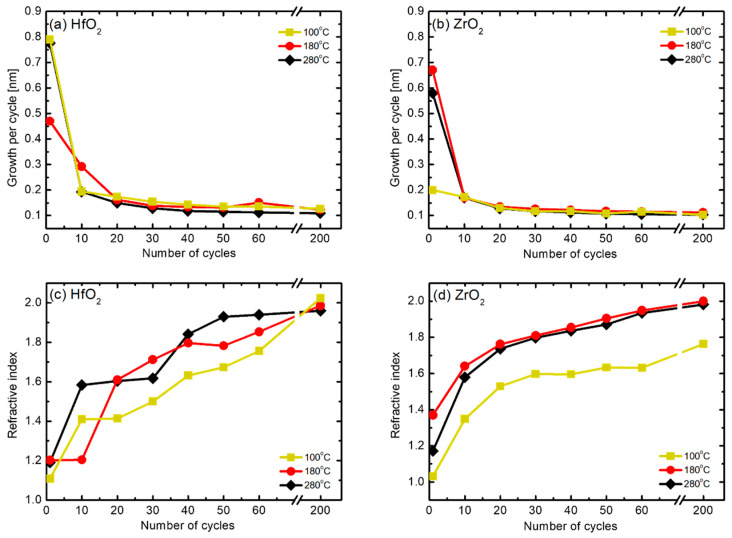
Changes in the (**a**,**b**) growth per cycle (GPC) and (**c**,**d**) refractive index of HfO_2_ and ZrO_2_ single thin films according to the number of cycles at different substrate temperatures.

Prior to the start of HZO thin film deposition, the growth conditions of single thin films of HfO_2_ and ZrO_2_ were confirmed. Each thin film showed self-limiting behavior when the source was injected for more than 2.5 s in the previous experiment. Accordingly, as shown in Table 1, the injection time of the source was set to 3 s to allow sufficient time. Figure 2 presents the results of analyzing the change in growth per cycle (GPC) and refractive index according to the number of cycles at various substrate temperatures. The results of GPC illustrated in Figure 2a,b show that the deposition thickness value is high in the initial cycles (that is, 10 cycles or less). This phenomenon is due to an overestimated measurement error that occurred during the planarization process because of the native roughness and curvature of the substrate at the initial stage; after 10 cycles, the thickness is almost constant regardless of temperature [24,25]. Furthermore, from the refractive index results shown in Figure 2c,d, a trend of change in the refractive index according to the number of cycles can be observed. Notably, the refractive index approaches 2.0 and 2.1, the bulk refractive index values of HfO_2_ and ZrO_2_, respectively, with an increase in the number of cycles at the different substrate temperatures. All the thin films deposited at 100 °C had a low refractive index, and the difference was particularly pronounced in the case of the ZrO_2_ thin film. It can be inferred from the Lorentz–Lorenz relation that the thin film density decreased at low deposition temperatures [26]. Based on the results in Figure 2, HfO_2_ and ZrO_2_ were deposited at rates of 0.123 nm and 0.112 nm per cycle, respectively, at the deposition temperature of 180 °C. The two materials were alternately deposited in each cycle, repeating the process 42 times, resulting in a Hf_0.5_Zr_0.5_O_2_ thin film with a thickness of approximately 10 nm.

**Figure 3:**
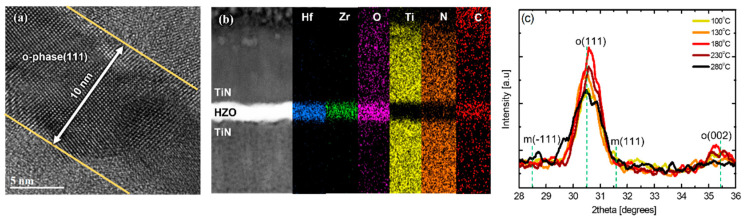
(**a**) Cross−sectional high−resolution TEM (HR−TEM) image and (**b**) EDS composition cross-sectional profile and (**c**) XRD pattern change with respect to substrate temperature in the range of 100–280 °C for PEALD HZO thin films deposited at 180 °C.

Figure 3a is a cross-sectional image obtained by the high-resolution TEM (HR-TEM) of a PEALD HZO thin film that was deposited at 180 °C and underwent post-annealing at 600 °C. The thickness of the thin film was approximately 10 nm, and an o-phase crystalline structure was mainly observed. The disappearance of the o-phase structure near the interface is thought to be because of interface instability due to TiN diffusion [13,27]. Figure 3b shows the EDS-based elemental composition profiles of the same thin film, and it can be observed that some of the Ti and N atoms diffused into the HZO thin film. In addition, nitrogen and carbon contamination was observed inside the HZO thin film owing to the TEMA precursors. In particular, the carbon contamination was considerable; herein, carbon is considered to be a residual impurity because the precursor is not completely decomposed during deposition [27,28,29,30]. Figure 3c shows the change in the XRD patterns of the PEALD HZO thin films according to the deposition temperature in the substrate temperature range of 100–280 °C. The peaks at 28.5 ° and 31.6 ° represent the m-phase, and the peaks at 30.5 ° and 35.4 ° represent the (111) and (200) planes of the o-phase [16]. At all deposition temperatures, the proportion of o-phase was greater than that of the m-phase, and it can be observed that the phase transformation to the o-phase was successfully achieved during the post-annealing at 600 °C. The intensity of the XRD peak corresponding to the o-phase was the highest at 180 °C, and it decreased as the deposition temperature decreased or increased further. In particular, in the case of deposition at a high temperature of 280 °C, secondary phases such as the m-phase were included.

**Figure 4:**
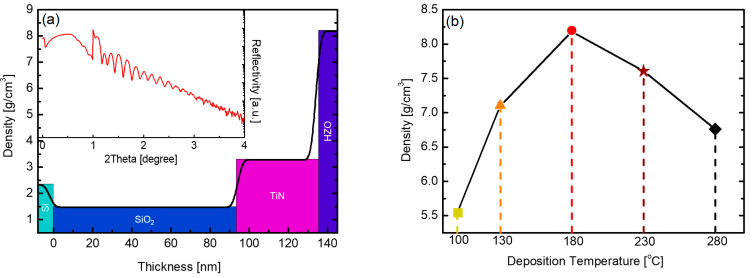
(**a**) XRR data of PEALD HZO thin film deposited at 180 °C and (**b**) HZO thin film density according to substrate temperature in the range of 100–280 °C.

Figure 4a presents the XRR data of the HZO thin film deposited at 180 °C, and the inset graph corresponds to raw data showing the reflectivity according to the X-ray incident angle. The density of the thin film is calculated based on the initial angle at which the reflectivity decreases. The thickness of the thin film is simulated through the oscillation period, and the thickness and the density of the deposited thin films constituting the sample can be calculated as shown in the outer graph. Figure 4b outlines the density change according to the substrate temperature of the PEALD HZO thin film obtained by this method. The density of the thin film was the highest (8.18 g/cm^3^) at the substrate temperature of 180 °C. This density exceeds the theoretical density of HZO [31]. In a multilayered structure, the density of each thin film is calculated by the reflectivity at each interface, but an error may occur if the thin film is too thin or the interface is not distinct. In this study, the deposited thin films were compared according to calculated density.

The density gradually decreased as the deposition temperature decreased or increased further. This trend is consistent with that of the o-phase peak intensity, as observed in the XRD patterns in Figure 3c. The decrease in density at low and high temperatures can be explained by the equation of Langmuir’s adsorption isotherm [32]. θ, the fraction of the surface covered by the absorbate, can be expressed as a function of time as shown in Equation (1).

(1) $$\frac{d\theta }{dt}=\gamma_{a}P_{i}\left(1-\theta \right)-\gamma_{d}\theta$$

Here, $\gamma_{a}$ denotes the adsorption coefficient, $\gamma_{d}$ is the desorption coefficient, and $P_{i}$ is the pressure. Here, the adsorption coefficient and the desorption coefficient are exponentially proportional to the temperature with respect to the activation energy required for adsorption and desorption, respectively.

(2) $$If\;\frac{d\theta }{dt}=0\;\left(equilibrium\right),\;\theta =\frac{P_{i}}{P_{i}+\gamma_{d}/\gamma_{a}}$$

Based on the assumption that the same pressure process applies in an equilibrium state where the adsorption rate becomes 0, if $P_{i}$ is set to a constant value, the equilibrium value of θ is highly dependent on temperature, as shown in Equation (2). At low temperatures, the value of $\gamma_{a}$ becomes small; consequently, sufficient chemisorption does not occur, and the space where adsorption does not occur remains empty. Furthermore, as θ decreases, the density of the thin film decreases. At high temperatures, $\gamma_{d}$ increases, and it is thought that owing to the empty space formed by the atoms desorbed during the deposition process, both the values of θ and the density of the thin film decrease. Under the conditions of this experiment, the substrate temperature of 180 °C results in the minimum $\frac{\gamma_{d}}{\gamma_{a}}$ value and the highest fraction of the surface covered by the absorbate; therefore, this condition is thought to be the optimal deposition condition that yields the highest density. In addition, the secondary phases, including the m-phase, appearing at a substrate temperature of 280 °C may cause density reduction [33,34].

**Figure 5:**
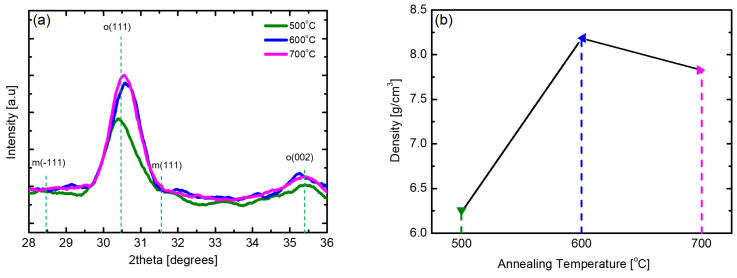
(**a**) XRD pattern and (**b**) density according to post-annealing temperature of PEALD HZO thin films deposited at 180 °C.

Although the m-phase is the most stable phase of HZO thin films, the formation of the m-phase is suppressed while the ratio of the ferroelectric o-phase is increased because of thermal stress caused by the difference in the thermal expansion coefficient between the HZO thin film and the TiN electrode during the post-annealing process [35,36]. Figure 5 shows the changes in the XRD patterns and density of samples of the HZO thin films deposited at 180 °C, the optimal substrate temperature, which underwent post-annealing at 500 to 700 °C. At all annealing temperatures, HZO thin films almost purely consisting of o-phases without the m-phase and secondary phases were obtained. In the case of the sample obtained via 500 °C post-annealing, the X-ray peak intensity was slightly weak, but in the samples obtained via post-annealing at 600 °C or higher, the X-ray peak intensity was strong. With regard to the density, the sample annealed at 600 °C showed the highest value; thus, the optimum annealing temperature was determined to be 600 °C. It was confirmed that the HZO thin film was densified, with crystallization, through the post-annealing process. It can be inferred from the results shown in Figure 5b that the density of the thin film may decrease as the interdiffusion between the TiN electrode and the HZO thin film increases under high-temperature post-annealing conditions.

**Figure 6:**
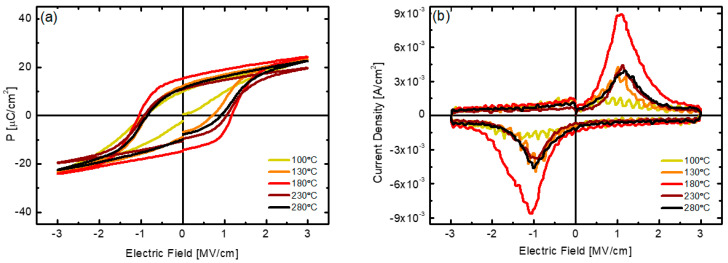
(**a**) P−E hysteresis curve and (**b**) polarization switching current curve with respect to electric field of HZO thin films deposited at various substrate temperatures.

The polarization characteristics of PEALD HZO thin films deposited at various substrate temperatures were evaluated. Figure 6a shows the PE hysteresis curves measured after 10^5^ cycles for each sample in which wake-up had occurred and the value of coercive field (2E_c_) was stabilized. Figure 6b shows the dynamic polarization switching current with respect to the electric field. The value of remanent polarization 2P_r_ measured based on each P-E hysteresis curve increased significantly from 12 μC/cm^2^ to 38.2 μC/cm^2^ as the deposition temperature increased from 100 °C to 180 °C. Further, as the strength of the coercive field (2E_c_) increased from 1 MV/cm to 1.97 MV/cm, the total area of the hysteresis curve increased significantly. Thereafter, as the deposition temperature increased to 280 °C, both the 2P_r_ and 2E_c_ values decreased, and this trend was consistent with the X-ray intensity of the o-phase and the density of the thin film. The maximum remanent polarization value of 38.2 μC/cm^2^ of the sample obtained at the deposition temperature of 180 °C is higher than the values reported in previous papers [14,16,19]. In Figure 6b, the maximum polarization current is observed near the coercive field. In the sample obtained at a deposition temperature of 180 °C, a dynamic switching current density of up to 8.8 × 10^−3^ A/cm^2^ was measured in an electric field of 1 MV/cm, and an almost symmetrical current pattern was also observed in negative electric fields. The trend of the polarization characteristics according to the deposition temperature is thought to be related to an increase in defects inside the HZO thin films deposited at low and high temperatures, as discussed above. These defects can limit the growth of the grain size and cause pinning of the switching of the ferroelectric domain under the external electric field [37,38,39,40].

**Figure 7:**
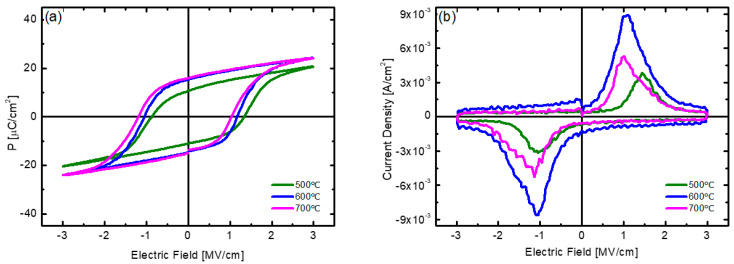
(**a**) P−E hysteresis curve and (**b**) polarization switching current curve with respect to electric field of HZO thin films deposited at substrate temperature of 180 °C with different post-annealing temperatures.

Figure 7 shows the results of analyzing the electrical properties of the HZO thin films deposited at 180 °C with post-annealing at various temperatures. The best 2P_r_ value and the largest dynamic switching current density were obtained at an annealing temperature of 600 °C. The HZO thin film annealed at 500 °C showed a 2P_r_ value of 21.6 μC/cm^2^ and a dynamic switching current density of up to 3.85 × 10^−3^ A/cm^2^, showing inferior characteristics compared to those of the samples annealed at 600 °C or higher. In addition, the P-E hysteresis curve and polarization switching current curve show asymmetry according to the sign, which indicates that a built-in potential is formed inside the thin film. From this, it can be inferred that at a low post-annealing temperature of 500°C, the distribution of defects inside the film was not symmetrical or that the phase change to the o-phase was not fully completed inside the thin film [41,42].

**Figure 8:**
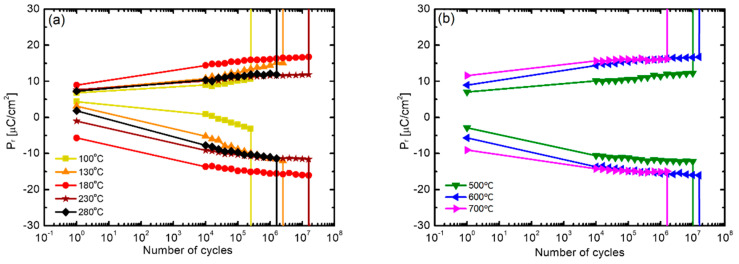
(**a**) Comparison of fatigue endurance of HZO films fabricated at different deposition temperatures after 600 °C annealing and (**b**) comparison of fatigue endurance of HZO films, deposited at a substrate temperature of 180 °C, with respect to annealing temperature.

Figure 8 compares the results of fatigue endurance evaluation of the HZO thin films according to the above-mentioned (a) deposition temperature and (b) post-annealing temperature. In Figure 8a, the HZO thin films deposited at 180 °C and 230 °C showed the highest level of endurance of 1.6 × 10^7^ cycles. The cases of deposition at the lowest temperature of 100 °C and the highest temperature of 280 °C showed relatively low endurances of 2.5 × 10^5^ cycles and 1.6 × 10^6^ cycles, respectively. In addition, in the case of these two samples, a wake-up phenomenon, in which the 2P_r_ value increased with the number of cycles, was clearly exhibited. Fatigue endurance is caused by the accumulation of impurities and oxygen vacancies inside the thin film at the electrode interface or crystal defects, and it is reported that the wake-up effect occurs in the process of redistribution of oxygen vacancies according to the application of an electric field [43,44]. It is inferred that samples with low density in the previous experiment contain many defects, resulting in low fatigue endurance or a marked wake-up effect. Figure 8b shows the results according to the annealing temperature, and it can be observed that the endurance of the thin film annealed at 600 °C is the highest. In addition, as the annealing temperature increases, the wake-up effect is improved; notably, the sample annealed at 700 °C shows the best improvement of the wake-up effect. However, the sample annealed at 700 °C showed the lowest fatigue endurance (1.6 × 10^6^ cycles), which is attributed to diffusion at the electrode interface [45,46].

The preparation methods and electrical properties of HZO films are summarized in Table 3 to compare our work with previous studies. The HZO thin film prepared at optimized PEALD conditions in this study showed relatively good remanent polarization and fatigue endurance performances despite being under the lowest deposition temperature.

In the case of the thin film deposited at the substrate temperature of 280 °C in the previous experiment, the presence of secondary phases such as the m-phase was confirmed. Therefore, it can be inferred that for increasing the ratio of the o-phase in HZO thin films, low-temperature deposition is advantageous. However, the density was greatly reduced in the thin films deposited at low temperatures. Therefore, this study investigated process improvement methods that can increase the density of thin films deposited at low temperatures. Four types of process improvement experiments (A to D) were performed for deposition at a substrate temperature of 100 °C, followed by RTA at 600 °C, and each process is outlined as follows. Process A is the process of improving θ, the fraction of the surface covered by the absorbate, by using the discrete feeding method (DFM). In the DFM, a purge step was included in the source injection step to refine the process, thereby removing the impurities and byproducts in the precursor injection step. This increased the initial chemisorption efficiency and fraction of the surface covered by the absorbate. In this experiment, the purge step was executed twice in the middle of the process. In process B, the plasma discharge time and oxygen injection time were increased by 4 s each; thus, the discharge time in this experiment was 6 s, and the oxygen injection time was 8 s. In process C, the total flow rate in the chamber was increased from 600 sccm to 900 sccm while maintaining the pressure. Finally, in process D, all of the aforementioned process improvement methods (A to C) were applied in combination.

**Figure 9:**
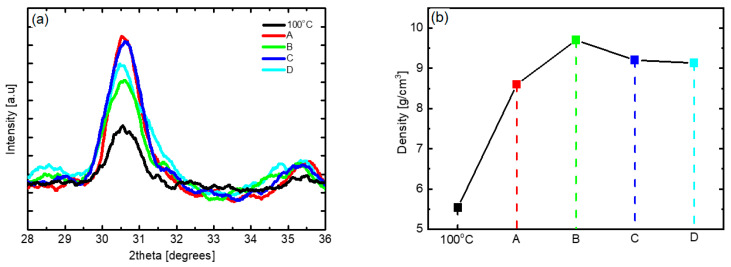
(**a**) XRD patterns and (**b**) thin film density according to the process improvement method of low-temperature-deposited HZO thin films; A: use of discrete feeding method, B: increase in RF plasma time, C: increase in gas flow, D: combined application of A, B, and C.

Figure 9 shows the changes in the XRD patterns and thin film density of the HZO thin films obtained with the various process improvement methods. In the results corresponding to processes A to D, both the o-phase peak intensity and the thin film density are significantly higher than those of the HZO thin film deposited at 100 °C without applying the process improvement. The crystallinity and density of the thin film improved upon applying process A. Specifically, θ, the fraction of the surface covered by the absorbate, increased because the application of the DFM eliminated unnecessary physical adsorption, thus stably providing the chemical adsorption sites to the precursor [25]. By applying process B, the density was significantly improved to 9.7 g/cm^3^, which is thought to be due to the reduction of oxygen vacancies based on the increase in the reaction time corresponding to the precursor-reactant reaction [20,47]. The results of process C confirmed that both the crystallinity and density were improved, and it is believed that the reduced boundary layer and increased diffusion rate due to the increase in the flow rate of the precursor enhanced the fraction of the surface covered by the absorbate of the precursor [48,49]. For process D, the o-phase peak intensity and thin film density were lower than those of processes A and C, which is inferred to be due to the interaction between process parameters. The results confirmed that by applying these process improvement methods, the θ for low-temperature deposition can be improved, and with an increased RF power supply time to promote the reaction with oxygen radicals, the oxygen vacancies can be reduced, resulting in properties similar to those of thin films deposited at high temperatures.

**Figure 10:**
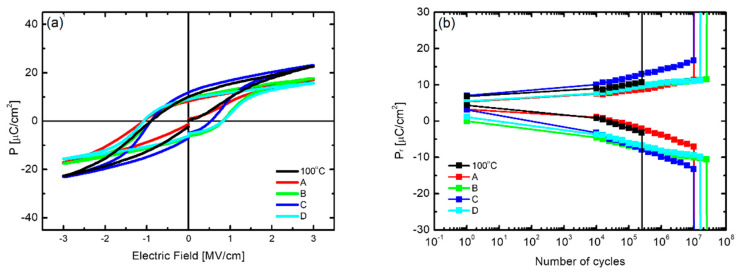
(**a**) P−E hysteresis curves and (**b**) fatigue endurance characteristics according to the process improvement method of low-temperature-deposited HZO thin films; A: use of discrete feeding method, B: increase in RF plasma time, C: increase in gas flow, D: combined application of A, B, and C.

The electrical properties of the low-temperature-deposited HZO thin films obtained with different process improvement methods were measured. Figure 10a shows the P-E hysteresis curves, and the HZO thin film obtained with process C showed the highest 2P_r_ value, 18.6 μC/cm^2^. Although this value was higher than the 2P_r_ value of 12 μC/cm^2^ of the thin film deposited at 100 °C without applying any process improvement, it was significantly lower than that of the thin film deposited at 180 °C. This is thought to be because although the physical properties of the thin films obtained by applying the process improvement methods were similar to those of the thin films deposited at 180 °C, the formation of a ferroelectric domain inside the thin films was hindered, and further investigation is needed to identify the cause. As shown by the fatigue endurance measurement results in Figure 10b, dielectric breakdown occurred at 10^7^ cycles or more, indicating that the service life was greatly improved through the process improvement. In particular, when process B was applied, the highest endurance of 2.5 × 10^7^ cycles was measured, which was superior to that of the thin film deposited at 180 °C. This increase in endurance is thought to be because the oxygen vacancies inside the thin film, which cause fatigue, were greatly reduced by the effects of the applied process improvement.

## 4. Conclusions

In this study, the characteristics and electrical properties of ferroelectric HZO thin films obtained by PEALD were evaluated according to the deposition temperature and annealing temperature. Further, we developed and applied various processes to improve these characteristics and electrical properties. First, since the growth per cycle (GPC) according to the deposition temperature of HfO_2_ and ZrO_2_ was constant, it was possible to deposit HZO thin films with similar deposition rates at all temperatures. The thickness of the deposited HZO thin films, o-phase crystalline structure, and elemental composition profiles were examined through cross-sectional TEM images and EDS analysis. The X-ray intensity of the o-phase of the thin film deposited at the substrate temperature of 180 °C was the highest, and mixed secondary phases such as the m-phase were observed in the thin film deposited at 280 °C. Density analysis of the thin films showed that the HZO thin film deposited at 180 °C had the highest density and a decrease in density was observed in the thin films deposited at temperatures lower and higher than 180 °C. To investigate the density change according to annealing temperature, the thin film samples were annealed in the temperature range of 500–700 °C, and a post-annealing temperature of 600 °C yielded the highest thin film density. The 2P_r_ value of the thin film fabricated with the deposition temperature of 180 °C and post-annealing at 600 °C was the highest, 38.2 μC/cm^2^, and the fatigue endurance was also the highest under these conditions, 1.6 × 10^7^ cycles. Three methods were proposed to enhance the density of the low-temperature-deposited thin films. For the low-temperature-deposited thin films with an increased RF plasma discharge time, the enhanced maximum density and an excellent fatigue endurance of 2.5 × 10^7^ cycles were obtained.

## Figures and Tables

**Table 1:** Conditions of HZO thin film deposition by PEALD and processes constituting one cycle.

Deposition Temperature Maintenance Gas Flow Pressure	100−280 °C 600 sccm 1.3 Torr
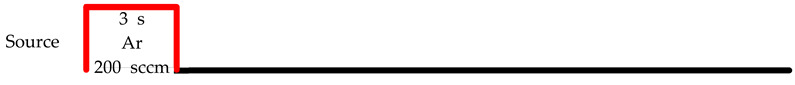	
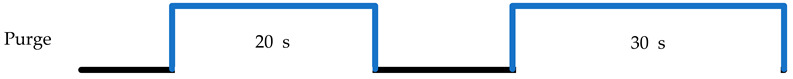	
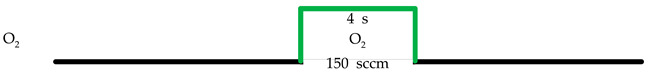	
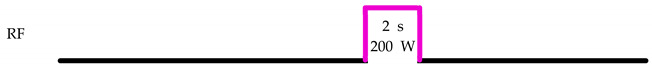	

**Table 2:** Conditions for rapid thermal annealing of HZO thin films after TiN top electrode deposition.

Annealing Temperature Ambient Pressure	500−700 °C N_2_ Atmosphere 5 Torr
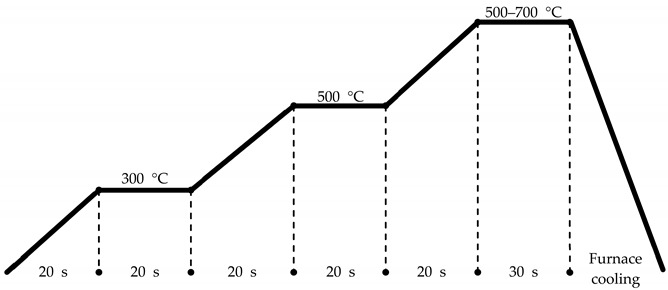	

**Table 3:** Summary and comparison of the preparation methods and electrical properties of HZO films.

Ref.	Growth Method	Electrode	Deposition Temperature	Annealing Temperature	2Pr (μC/cm^2^)	Fatigue Endurance (Number of Cycles)
Our work	PEALD	TiN	180 °C	600 °C	38.2	1.6 × 10^7^
[19]	PEALD	TiN	250 °C	450 °C	35	1.6 × 10^5^
[10]	THALD	TiN	280 °C	600 °C	29	−
[11]	THALD	Ru	280 °C	500 °C	36	1.2 × 10^11^
[12]	THALD	TiN	250 °C	400 °C	48	−
[13]	THALD	W	250 °C	720 °C	42	1 × 10^4^

## Data Availability

The data presented in this study are contained within the article.
